# Epitopes for neutralizing antibodies induced by HIV-1 envelope glycoprotein BG505 SOSIP trimers in rabbits and macaques

**DOI:** 10.1371/journal.ppat.1006913

**Published:** 2018-02-23

**Authors:** P. J. Klasse, Thomas J. Ketas, Christopher A. Cottrell, Gabriel Ozorowski, Gargi Debnath, Diawoye Camara, Erik Francomano, Pavel Pugach, Rajesh P. Ringe, Celia C. LaBranche, Marit J. van Gils, Christine A. Bricault, Dan H. Barouch, Shane Crotty, Guido Silvestri, Sudhir Kasturi, Bali Pulendran, Ian A. Wilson, David C. Montefiori, Rogier W. Sanders, Andrew B. Ward, John P. Moore

**Affiliations:** 1 Department of Microbiology and Immunology, Weill Cornell Medical College, New York, New York, United States of America; 2 Department of Integrative Structural and Computational Biology, IAVI Neutralizing Antibody Center and Center for HIV/AIDS Vaccine Immunology and Immunogen Discovery, The Scripps Research Institute, La Jolla, California, United States of America; 3 Department of Surgery, Duke University Medical Center, Durham, North Carolina, United States of America; 4 Department of Medical Microbiology, Academic Medical Center, University of Amsterdam, Amsterdam, The Netherlands; 5 Center for Virology and Vaccine Research, Beth Israel Deaconess Medical Center, Boston, Massachusetts, United States of America; 6 Division of Vaccine Discovery, La Jolla Institute for Allergy and Immunology, La Jolla, California, United States of America; 7 Scripps Center for HIV/AIDS Vaccine Immunology and Immunogen Discovery, La Jolla, California, United States of America; 8 School of Medicine, Division of Infectious Diseases, University of California, San Diego, La Jolla, California, United States of America; 9 Yerkes National Primate Research Center, Emory University, Atlanta, Georgia, United States of America; 10 Emory Vaccine Center/Yerkes National Primate Research Center at Emory University, Atlanta, Georgia, United States of America; 11 The Skaggs Institute for Chemical Biology, The Scripps Research Institute, La Jolla, California, United States of America; Miller School of Medicine, UNITED STATES

## Abstract

The native-like, soluble SOSIP.664 trimer based on the BG505 clade A *env* gene of HIV-1 is immunogenic in various animal species, of which the most studied are rabbits and rhesus macaques. The trimer induces autologous neutralizing antibodies (NAbs) consistently but at a wide range of titers and with incompletely determined specificities. A precise delineation of immunogenic neutralization epitopes on native-like trimers could help strategies to extend the NAb response to heterologous HIV-1 strains. One autologous NAb epitope on the BG505 Env trimer is known to involve residues lining a hole in the glycan shield that is blocked by adding a glycan at either residue 241 or 289. This glycan-hole epitope accounts for the NAb response of most trimer-immunized rabbits but not for that of a substantial subset. Here, we have used a large panel of mutant BG505 Env-pseudotyped viruses to define additional sites. A frequently immunogenic epitope in rabbits is blocked by adding a glycan at residue 465 near the junction of the gp120 V5 loop and β24 strand and is influenced by amino-acid changes in the structurally nearby C3 region. We name this new site the “C3/465 epitope”. Of note is that the C3 region was under selection pressure in the infected infant from whom the BG505 virus was isolated. A third NAb epitope is located in the V1 region of gp120, although it is rarely immunogenic. In macaques, NAb responses induced by BG505 SOSIP trimers are more often directed at the C3/465 epitope than the 241/289-glycan hole.

## Author summary

A protective vaccine would constitute a breakthrough in efforts to curb the global spread of HIV. Such a vaccine should induce antibodies inhibiting infection by most strains of the virus that circulate worldwide. Engineered SOSIP trimer mimics of the envelope glycoprotein on the surface of HIV particles, which mediates viral entry into cells, can elicit such neutralizing antibodies in rabbits and rhesus monkeys. These antibodies, however, have a narrow specificity, neutralizing mainly the same virus from which the SOSIP trimer protein was derived. Here, we have mapped the sites on the SOSIP trimer to which these antibodies bind, thereby delineating both an already identified binding site and a previously unknown one. The rabbits produced neutralizing antibodies that recognize both binding sites, but the rhesus monkeys responded predominantly to the newly identified one. As immune responses in monkeys are the more likely to resemble those in humans, the findings described here might aid strategies to steer human antibody responses to sites that are cross-reactive among HIV strains. That outcome would be a major step towards an effective vaccine.

## Introduction

The induction of neutralizing-antibody responses against human immunodeficiency virus type 1 (HIV-1) is the objective of vaccine programs based on various soluble envelope glycoprotein (Env) trimers (reviewed in [1–12]). The prototype native-like recombinant Env trimer is the BG505 SOSIP.664 construct, which is based on a clade A *env* gene [13–16]. This trimer, like many subsequent ones of similar design from multiple genotypes, mimics the native structure of the Env spikes on the surface of HIV-1 virions that are targeted by neutralizing antibodies, NAbs [15, 17–20]. Accordingly, native-like trimers such as BG505 SOSIP.664 are used in vaccine development programs aimed at eliciting NAbs, particularly those with broad activity against diverse HIV-1 strains (i.e., bNAbs). Although the BG505 SOSIP.664 and other native-like trimers express the epitopes for multiple bNAbs, they do not elicit such antibodies in rabbits or macaques [21–25]. It is well known that antigenicity does not equal immunogenicity: the presence of a bNAb epitope on an Env trimer does not ensure that NAbs will be raised against it [3, 4, 26–28].

It is difficult to test and compare many early generation immunogens in humans, because of the high costs and complex, time-consuming procedures required for producing clinical-grade proteins [29, 30]. To determine the immunogenicity of a trimer, antibody responses induced in animals are quantified and the targeted epitopes are mapped. To date, BG505 SOSIP trimers have most often been studied in rabbits and macaques. And, although macaques are genetically closer to humans, rabbits are less expensive and more readily available. In both species, the trimers induce NAbs against the autologous, neutralization-resistant (Tier-2) BG505.T332N virus, but generally not against heterologous Tier-2 viruses [21, 23–25, 31–34]. BG505 virus mutants have been used to map the autologous NAb epitopes in sera from trimer-immunized rabbits and, on a smaller scale, macaques [23–25]. Thus, we reported that a hole in the glycan shield of the BG505 virus, centered on residues S241 and N289, is a frequent target for autologous NAbs induced in rabbits, but we also inferred that a substantial minority of the immunized animals raised NAbs against additional, unknown epitopes [23]. Rabbit monoclonal antibodies (MAbs) against that 241/289-glycan-hole site have been characterized [35]. But little is known about the epitopes for autologous NAbs induced in trimer-immunized macaques. Some evidence suggests that the 241/289-glycan hole is targeted in a subset of macaques, although less often than in rabbits [24]. Here, we designed and used a much larger panel of BG505-*env* pseudo-viral mutants to delineate these known and unknown rabbit and macaque NAb epitopes.

We first selected a panel of 15 rabbit sera in which the autologous NAbs are known to target, or not, the 241/289-glycan-hole site [23]. The serum panel and the new *env* mutants enabled us to identify a second epitope cluster on BG505 Env trimers that is frequently targeted by rabbit NAbs. This site involves a stretch of the gp120 C3-region that is known to have been under selection pressure in the HIV-1-infected infant from whom the BG505 virus was isolated [13, 16]. The site is also blocked by knocking-in a glycan at residue 465, near the junction of the V5 loop with the β24-strand. Furthermore, two sequence changes in the gp120 V1-region affected neutralization by two of the 15 rabbit sera in the test panel, and probably identify a third NAb epitope. Several other sequence changes directly or indirectly affected how potently the rabbit sera neutralized the BG505 pseudo-virus. We then found that NAbs in sera from 15 BG505 SOSIP trimer-immunized macaques were predominantly directed at the C3/465 epitope cluster. Overall, the autologous NAb response to the BG505 SOSIP trimer is complex. As noted previously, it is not restricted to a single hole in the glycan shield at positions 241/289 [23]. Our refined knowledge of the targets for BG505 SOSIP trimer-induced NAbs may inform the design of new immunogens that are better able to elicit bNAbs.

## Results

### Source and choice of rabbit sera

**Fig 1:**
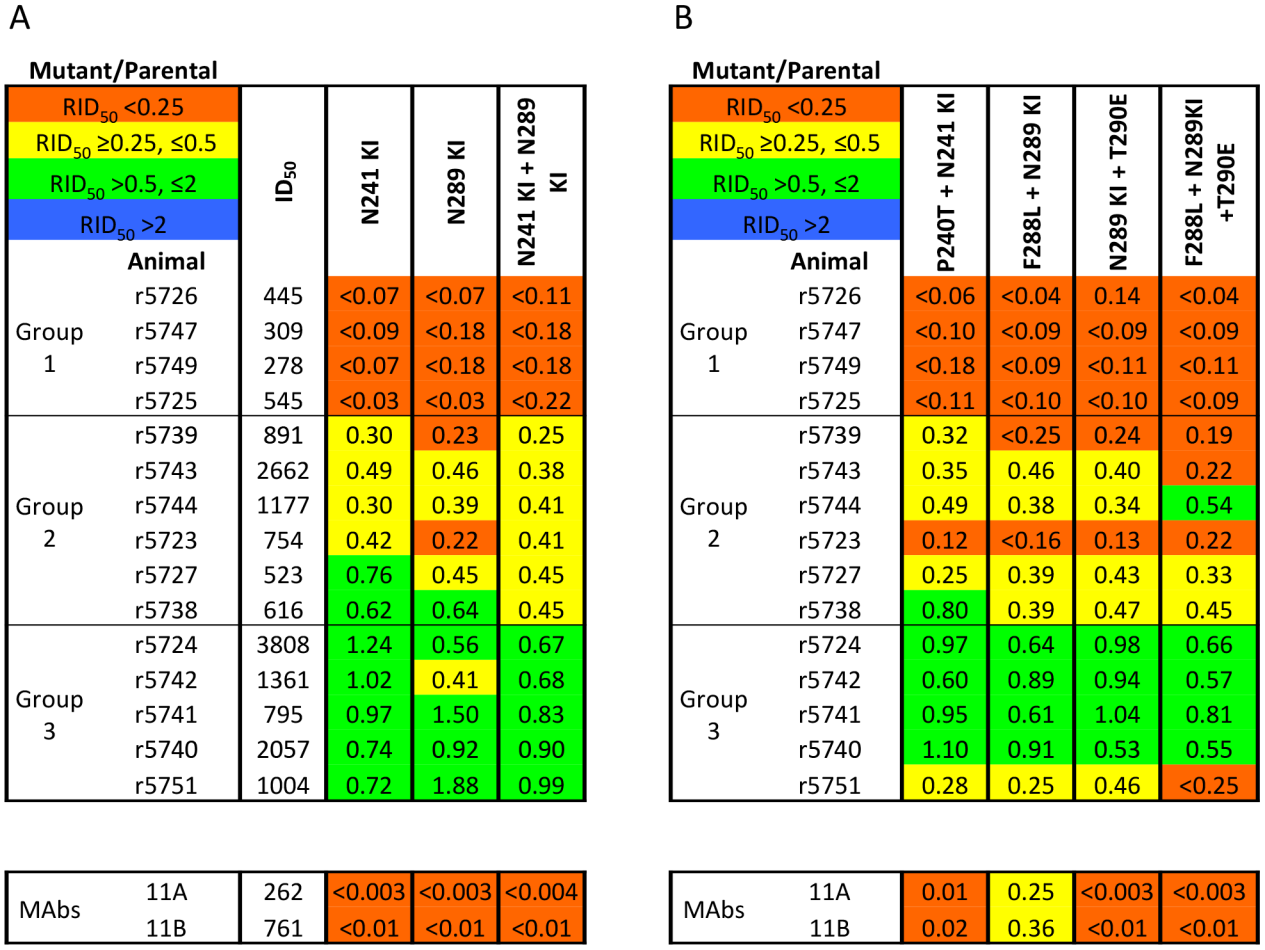
A panel of rabbit sera with a spectrum of sensitivities to glycan knock-in mutations at positions 241 or 289 or both. For each serum designated to the left, the ID_50_ values against each mutant pseudovirus relative to the BG505.T332N parental virus (RID_50_ = relative ID_50_, i.e., (ID_50_ against mutant)/(ID_50_ against parental)) are recorded, and then color-coded as outlined in the top-left key. Thus, mutations conferring insignificant, intermediate or strong neutralization resistance are in green, yellow or red, respectively. The Env mutations are summarized in the top row, with the complete sequence changes in the mutants listed in S1 Table. KI = glycan knock-in. Each tabulated value is the midpoint or median of ≥2 replicates. The sera are rank-ordered from the strongest to the weakest effect of the N241-KI/N289-KI mutations and divided into sub-groups: Group-1, complete neutralization resistance; Group-2, intermediate outcomes; Group-3, no effect on neutralization. **A**. The absolute inhibitory dilution factors against the parental virus (ID_50_) are given in the first data column. The single N241-KI and N289-KI mutants and the double N241-KI/N289-KI mutant had similar neutralization phenotypes within each of the three serum sub-groups. **B**. Non-glycan mutations that affect the lining of the 241/289-glycan hole, when combined with either the N241-KI or N289-KI changes, only sporadically increased resistance to the Group-2 or Group-3 sera.

The sera used in the present analysis were derived from a published experiment exploring the immunogenicity of BG505 SOSIP.664 trimers in rabbits [23]. There, we inferred that, while around half of the immunized rabbits produced NAbs targeting the 241/289-glycan hole on the BG505.T332N virus, the NAb response in the remainder recognized one or more unknown sites [23]. For more precise mapping, we therefore selected a panel of 15 high-titer sera that represent the spectrum of recognition of the 241/289-glycan hole (Fig 1A). Specifically, the 4 sera (r5726, r5747, r5749, r5725) in Group-1 represent the dominant subset in which neutralization is eliminated by the single and double N241/N289-glycan-knock-in (KI) changes, the 6 sera (r5739, r5743, r5744, r5723, r5727, r5738) in Group-2 have reduced titers against those 241/289-glycan-KI mutants, and the 5 sera (r5724, r5742, r5741, r5740, r5751) in Group-3 have intact titers against them (i.e., the NAbs target a different site). Note the sera in these three groups were chosen in order to identify unknown epitopes: the number of sera in each group (4 *vs*. 6 *vs*. 5) does not reflect the relative frequency of their neutralization profiles in a larger set of sera from trimer-immunized rabbits (see Discussion) [23]).

In Fig 1A, the sera are ranked (top to bottom) by their sensitivity to the N241-KI/N289-KI mutant, as reported by the ratio of the mutant over the parental ID_50_ values (i.e., the relative ID_50_, RID_50_, see Methods). We found no strict relationship between immunization history and the serum neutralization titers against the N241-KI/N289-KI mutants (BG505 only vs. BG505 and B41 immunizations, RID_50_ for N241-KI, p = 0.79; N289-KI, p = 0.16; N241-KI/N289-KI, p = 0.42) but we detected marked correlations between these titers and the absolute titers against the parental BG505.T332N pseudo-virus (N241-KI, r = 0.63, p = 0.014; N289-KI, r = 0.60, p = 0.020; N241-KI/N289-KI, r = 0.63, p = 0.015 (Fig 1A). Hence, although the 241/289-glycan hole is the dominant target for NAbs in many rabbit sera, NAbs against the yet uncharacterized subdominant epitopes are more potent or present at higher concentrations.

We tested the 15 selected sera against the parental and 141 BG505.T332N mutant viruses (plus two clones and one mutant derived from the maternal MG505 virus, S1 Table) and then analyzed the clustering among the ranked sera of effects on neutralization. The relative neutralization titers of 50 glycan-knock-out (KO) or knock-in (KI) (S1 Fig) and 55 non-glycan (S2 Fig) mutants are shown in the SI. The resulting 1575 serum-virus combinations yielded sporadic examples of increased or decreased neutralization titers. The generally null outcomes of this large body of tests strengthen the significance of what was seen with the selected virus mutants that are discussed below.

In Fig 1 and subsequent figures with rabbit data we also show the effect of the mutations on neutralization by two MAbs, 11A and 11B, which were previously isolated from BG505 trimer-immunized rabbits and target the 241/289-glycan hole [35]. These results are described separately below.

### Mutations that affect the 241/289-glycan hole or have similar effects

First, we investigated whether adding mutations of residues nearby the N241-KI or N289-KI changes (P240T, F288L and T290E) created viruses that were more strongly resistant to the Group-2 or resistant at all to Group-3 sera (Fig 1B). Additional reductions in neutralization titers were rare, and the multiply mutated viruses were still neutralized potently by 4 out of 5 Group-3 sera. We infer that some NAbs in Group-2 sera and most in Group-3 are directed at epitopes elsewhere on the trimer.

**Fig 2:**
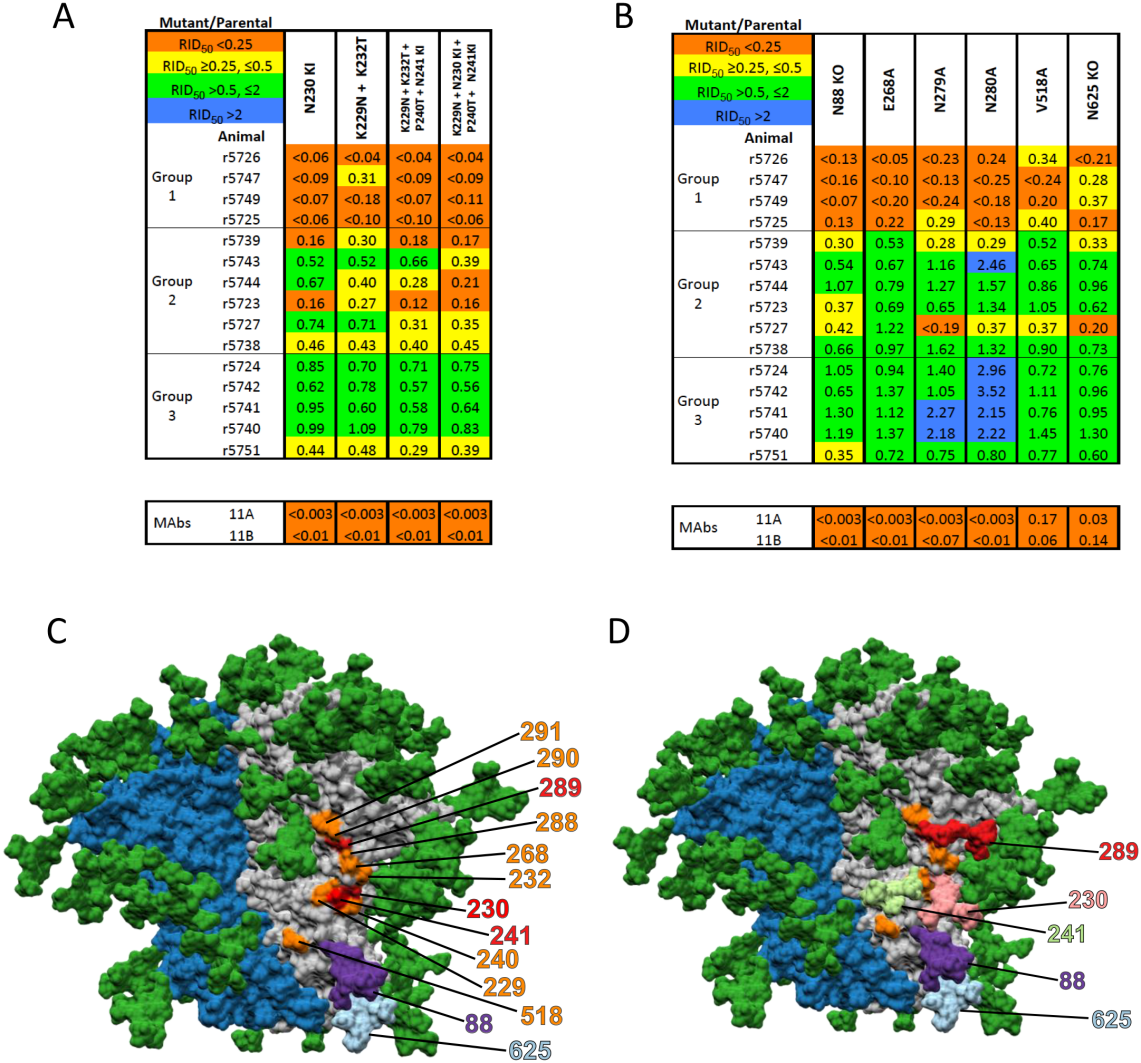
Mutations with effects similar to those of the N241-KI and N289-KI changes on neutralization by rabbit sera. The layout in panels **A** and **B** mirrors that of Fig 1A. **A**. The N230-KI mutation ablated neutralization by Group-1 sera and had an intermediate effect on Group-2 sera. Combining mutations around residue D230 with the N241-KI and the P240T mutations somewhat expanded the neutralization-reducing effects in Groups-1 and -2. **B**. Changes in C2 and the N88-KO or N625-KO mutations reduced neutralization by Group-1 sera, and hence resembled the N241-KI and N289-KI mutations. **C**. A surface-rendered model of the BG505 SOSIP.664 trimer seen from the side with the apex up. The peptidic surface on the protomer with marked residues is grey and that on the neighboring protomer is dark blue; the peptidic surface of the third protomer is not seen. The glycans are dark green. The D230 and S241 and N289 residues are highlighted in red and other residues involved in the epitope are in orange. The glycans at positions N88 and N625 are colored in purple and light blue, respectively. **D**. The model indicates the predicted orientations of the glycans present on the N230-KI (pink), N241-KI (light green) and N289-KI (red) mutants; the N88 and N625 glycans are also colored, as in panel-C. The model is based on PDB-5V8M.

We next identified mutants with neutralization profiles resembling those of the N241-KI and N289-KI mutants (Fig 2A and 2B, compare with Fig 1A). Two of these were the N230-KI mutant and a double mutant, K229N/K232T, which involves the two adjacent residues but without adding a glycan site (Fig 2A). Combining the K229N/K232T changes with the P240T and N241-KI mutations, either with or without the N230-KI mutations, had little additional effect: in particular, the potencies of the Group-3 sera against these combination-mutant viruses were unchanged for 4 out of 5 rabbits (Fig 2A).

Two glycan-KO mutants, N88-KO and N625-KO, and 4 non-glycan point mutants, E268A, N279A, N280A and V518A, were usually strongly resistant to Group-1 sera but sensitive to at least 4 out of 5 Group-3 sera (Fig 2B, again compare with Fig 1A). In addition, the two mutations at adjacent residues, N279A and N280A, increased the neutralization potency of multiple Group-3 sera. The distinctive profiles of specific mutants again suggest that NAb specificities differ among the three serum sub-groups.

Structural modeling showed that the glycan added by the N230-KI mutation could project into the 241/289-glycan hole epitope and potentially restrict antibody access to it (Fig 2C). (Fig 2C). The model also indicated that residue E268 is located in the center of the 241/289-glycan hole, which accounts for the impact of its mutation (Fig 2C). Residue V518, in the gp41 fusion peptide (FP), is further away, but a substitution here could indirectly affect the bottom of the glycan-hole epitope (Fig 2C). Alternatively, some FP residues might directly contribute to an extended epitope centered on the 241/289-glycan hole. The predicted orientations of the glycans knocked-in at positions 230, 241 and 289 are shown in Fig 2D.

### Mutations that identify a new NAb epitope: The C3/465 epitope

**Fig 3:**
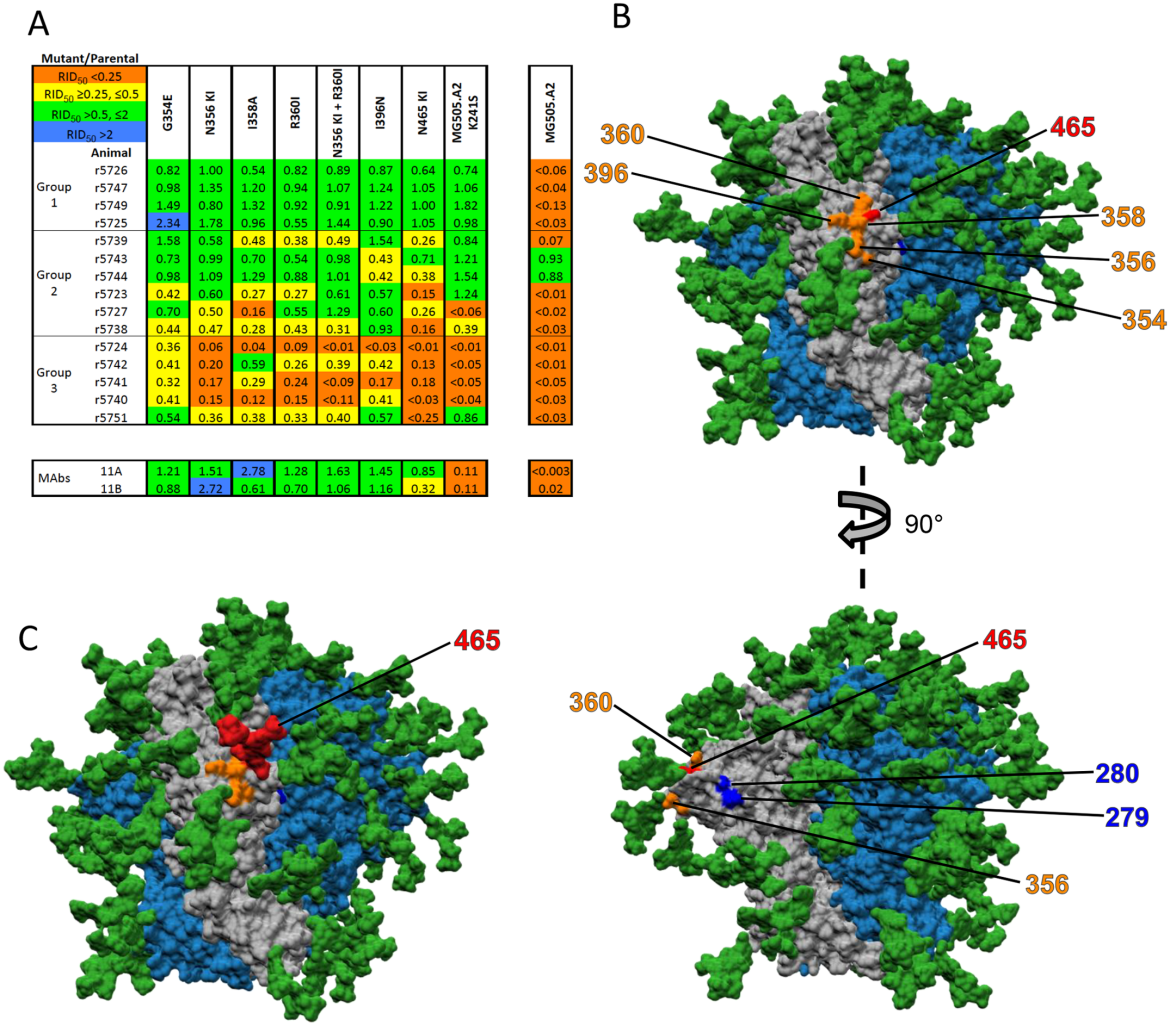
Mutations identifying the C3/465 epitope as a new rabbit NAb epitope. **A**. The layout is the same as in Fig 1. Specific mutations in C3 and V4, including N465-KI, conferred resistance to Group-3 and some Group-2 sera, but not to Group-1 sera; their effect was therefore the converse of the N241-KI and N289-KI changes. The K241S mutant of the MG505 cl.A2 virus had a very similar profile (compare the mutant with the parental clone shown in a separate column to the right, and see also Fig 4; all RID^50^ values are relative to the BG505.T332N parental virus). **B**. A surface-rendered model of the BG505 SOSIP.664 trimer seen from the side with the apex up. The peptidic surface on the protomer with marked residues is grey and those surfaces on the neighboring protomers are dark blue. The glycans are dark green, as in C. The residues G354, N356, I358, R360 and I396 are highlighted in orange and T465 in red; the upper and lower panels show the same model, but from two perspectives rotated by 90°. In the lower panel, residues N279 and N280 are also highlighted, in blue. **C**) Structural model indicating the predicted orientation of the glycan (in red) now present on the N465-KI mutant, with residues contributing to the C3/465 epitope in orange.

A set of BG505.T332N mutants were generally resistant to the Group-3 but sensitive to the Group-1 sera, i.e., sensitive to NAbs targeting the 241/289-glycan hole (Fig 3A). The N465-KI mutant (derived from MG505 cl.H3), which has a glycan knocked-in at residue 465 at the junction between the V5 loop and the β24 strand, was strongly discriminatory. Other mutations at residues nearby in the primary structure had sporadic positive and negative effects on neutralization but lacked this discriminatory neutralization profile (N461-KI, N462-KO, see S1 Fig). For the full panel of 15 sera, the relative titers against the N241-KI/N289-KI mutant correlated inversely but strongly with those against the N465-KI mutant (r = -0.78, p = 0.0010). This inverse correlation supports what is apparent from a visual comparison of Figs 1A and 3A; the comparison also indicates that residue T465 is involved in the NAb-epitope cluster recognized by Group-3 sera, which is distinct from the 241/289-glycan hole target for Group-1 (Figs 1A, 2A, 3A and 3B).

The C3 region of gp120 is under selection pressure during human infection with the BG505 virus [13, 16], and we had found that this region influences neutralization by sera from BG505 trimer-immunized rabbits [25]. Accordingly, we designed a set of C3-mutant viruses. Several of them, including G354E, N356-KI, I358A, R360I and N356-KI/R360I, had neutralization profiles similar to that of the N465-KI mutant, suggesting a linkage among them (Fig 3A). Structural modeling showed that these C3 residues and residue T465 are clustered, and that an N465-KI mutation could plausibly occlude access to an epitope (Fig 3B and 3C). Thus, we describe a new BG505 NAb site that we term “the C3/465 epitope.”

A key role for residues I358 and R360 in the C3/465 epitope was further emphasized by the neutralization profile of the K241S mutant of the maternal MG505 cl.A2 virus, which closely resembled the profiles of the C3/465 mutants (RID_50_ values for both the clone and mutant in relation to the comparator BG505.T332N virus are given in Fig 3A). The cl.A2 virus was generally resistant to the panel of 15 sera, but its K241S mutant was fully sensitive to all 4 Group-1 sera, to 4 of the 6 Group-2 sera and to one Group-3 serum (Fig 3A). Thus, the loss of the bulky lysine chain at position 241 permits NAb binding to the epitope in the 241/289-glycan hole, but does not confer sensitivity to the C3/465-directed NAbs. Furthermore, we noted that the only differences between the MG505 cl.A2 and BG505.T332N viruses among the residues listed in Fig 3A are at positions 358 and 360. Specifically, the N356-KI (I358T) and R360I mutations introduce the cl.A2 residues into the BG505.T332N virus. The resulting single and double mutants were generally resistant to Group-3 sera (Fig 3A). We consider it relevant that these two neutralization resistance-associated sequence differences arose naturally between the maternal and infant viruses [13, 16]. Two further C3-region mutants, G354E and N356-KI, behaved similarly to the others that define the C3/465 site. Based on the neutralization and structural analyses, we propose that the new NAb epitope cluster is centered on residues I358 and R360 and includes a small glycan hole lined by residue T465, which is adjacent to the N462 glycan (Fig 3B). The I396N substitution also preferentially reduced the titers of Group-3 sera, although only partially (Fig 3A). The spatial proximity of residue I396 in V4 to residue I358 in C3 explains its impact, while the weaker effect of the G354E mutation suggests that this residue is located at the periphery of the epitope (Fig 3B).

Neutralization titers of Group-2 sera were reduced by the mutations affecting either epitope cluster (Figs 1A, 2A and 3A); one serum in Group 3, r5751, although potent against the N241KI/289KI mutants, resembled Group 2 in other aspects (Figs 1B, 2A, 2B and 3). These sera may contain NAbs against both parts of an extended 230/241/289-glycan-hole epitope cluster and the newly identified C3/465 epitope. Alternatively, individual NAbs in these sera recognize an epitope cluster that is affected by mutations at either of these two sites, although it is not obvious from the structural models where it could be located.

### Effects of V1 mutations on neutralization

**Fig 4:**
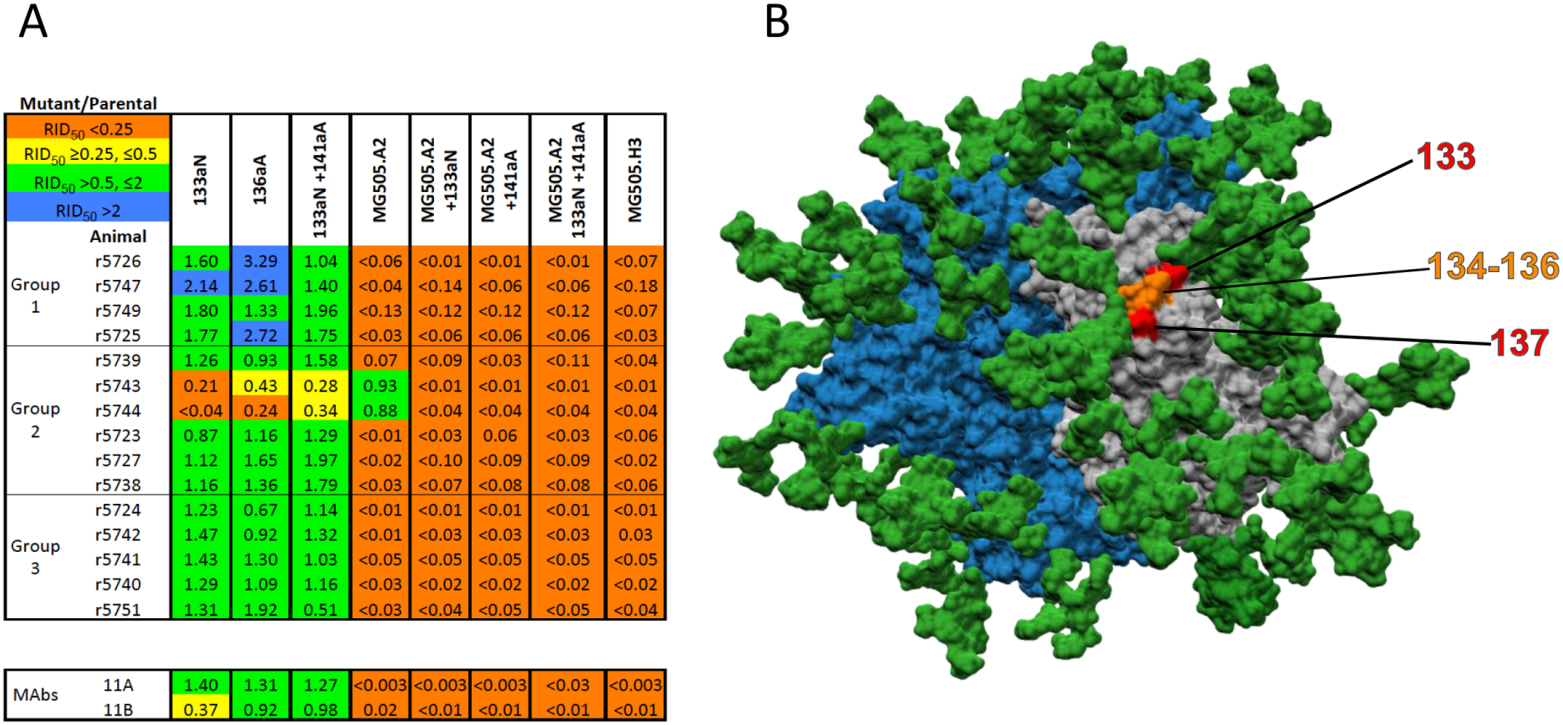
V1 mutations conferring neutralization resistance to two rabbit sera. **A**. The layout is the same as in Fig 1. No serum neutralized the MG505 cl.H3 virus but two Group-2 sera did neutralize MG505 cl.A2. Inserting the two V1 residues (133aN and 136aA), present in cl.H3 but absent from cl.A2, into the parental BG505.T332N virus, alone or together, conferred resistance to only those two sera. **B**. An oblique view of the apex of the BG505 SOSIP.664 trimer with the peptidic surface of the protomer with marked residues colored in grey and those surfaces on the other two protomers colored in dark-blue. The glycans are dark green. The positions of V1 residues 133 and 137 are shown in red and 134–136 in orange.

Two V1-region mutants and the corresponding double mutant of BG505.T332N were largely resistant to the Group-2 sera r5743 and r5744 (Fig 4A). The 133aN and 136aA mutations (derived from MG505 cl.H3) insert single residues in V1; 133aN moves the N133- glycan site one position C-terminally (S1 Table). The local V1 sequence in the parental BG505.T332N virus is TNVTNN and in the 133aN/136aA double mutant it is TN**N**VTN**A**N (the two inserted residues are highlighted in bold and underlined). The same two sera were also the only ones able to neutralize MG505 cl.A2 (the RID_50_ values relative to BG505 T332N are given in Fig 4A). The MG505 cl.H3 virus, which was not neutralized by any of the 15 sera, differs from cl.A2 in having the 133aN and 136aA insertions (Fig 4A). And when these cl.H3-derived 133aN and 136aA insertions were introduced into the cl.A2 V1 region, the resulting mutants resembled cl.H3 in being fully resistant to sera r5743 and r5744 (Fig 4A). Likewise, when introduced into BG505 T332N comparator, they conferred resistance only to these two sera. Taken together, these data provide evidence for a NAb epitope somehow involving V1 residues N133 and N136. We also note that neutralization of the BG505.T332N virus by the r5743 and r5744 sera was little affected by the N465-KI and related C3 mutations, reinforcing the argument that these two sera are targeting an atypical epitope in addition to the 241/289-glycan hole (Fig 3). The V1 neutralization epitope, however, appears to be inconsistently immunogenic, as only these two Group-2 sera among the 15 tested were affected by the 133aN and 136aA mutations. The structural locations of the V1 residues are shown in Fig 4B.

### Pan-resistance mutations

**Fig 5:**
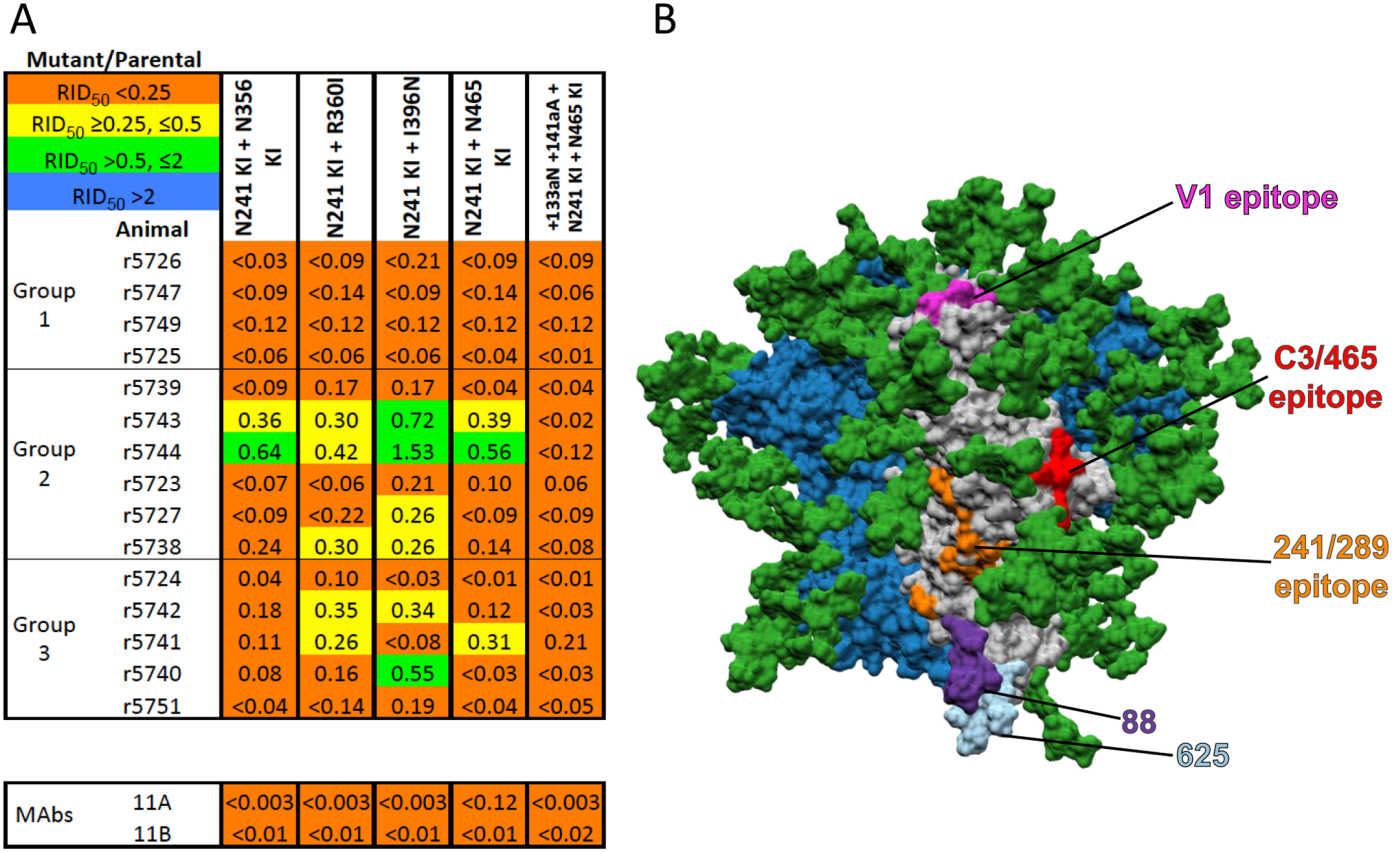
Pan-resistance mutations that markedly affect nearly all rabbit sera. **A**. The layout is the same as in Fig 1A. Combining the N241-KI mutation with the N465-KI or other point mutations that had correlated effects (e.g., those in C3) strongly reduced neutralization by all three sub-groups of sera. **B**. A surface-rendered model of the BG505 SOSIP.664 trimer seen from the side with the apex up. The peptidic surface on the protomer with marked residues is grey and those surfaces on the neighboring protomers are dark blue. Glycans are dark green. The 241/289-glycan hole epitope (orange), The C3/465 epitope (red) and the V1 epitope (magenta) are highlighted. The N88 and N625 glycans are also colored, in purple and light blue respectively.

The above evidence is consistent with the existence of three autologous NAb epitopes on BG505 Env trimers. To strengthen the argument, we designed virus mutants on which the epitopes were simultaneously mutated. Some of the resulting combination mutants, particularly those including the N289-KI mutation, were poorly infectious and were not studied further, but we identified five that could be tested against the panel of 15 sera. Specifically, combining the N241-KI mutation with N465-KI or with the N356-KI or R360I changes in C3 or with I396N in V4 created viruses that were generally and strongly resistant to all three sub-groups of rabbit sera (Fig 5A). Of note is that the two Group-2 sera with specific sensitivity to V1 changes, r5743 and r5744, had moderately reduced titers against the above combination mutant viruses, but they did not neutralize the N241-KI/N465-KI double mutant when the 133aN and 136aA V1 insertions were added (Fig 5A). Again, this finding supports the existence of a NAb epitope that includes or is influenced by the stretch of V1 residues around positions N133 and T136. The locations of the three autologous NAb epitopes on the BG505 SOSIP.664 trimers are depicted in Fig 5B.

### Comparison of mutant neutralization by polyclonal and monoclonal rabbit antibodies

As positive controls for sensitivity to 241/289-KI mutations, and as tools for determining the polyclonal NAb specificities in the sera, we used two rabbit MAbs, 11A and 11B, which have been reported to target the 241/289-glycan hole [35]. The neutralization activity of both MAbs was eliminated by the N241-KI and N289-KI mutations and by almost all the other changes that predominantly affected the Group-1 sera (Figs 1 and 2). The potency of MAb 11B was, however, also somewhat reduced by the N465-KI mutation that predominantly affected neutralization by Group-3 sera (Fig 3A). In contrast, the C3 mutations that reduced the titers of the Group-3 sera did not reduce the neutralization potency by either MAb; the N356-KI and I358A mutations increased their potencies. Unlike the Group-1 and most group-2 sera, MAbs had markedly reduced potency against the cl.A2 K241S mutant. The V1 mutation 133aN reduced neutralization by MAb 11B, whereas the V1 mutants sporadically enhanced the activity of Group-1 sera (Fig 4A). Finally, neither MAb neutralized the viruses with the pan-resistance mutations (Fig 5A).

The MAb epitope mapping study yields several insights. First, the reductions in serum neutralizing titers caused by multiple, widely dispersed mutations cannot necessarily be attributed to antibody poly-specificity. For the MAbs also had complex neutralization profiles that could only be explained by a combination of direct and indirect effects of some mutations on their epitopes. Second, since the epitopes of these MAbs and their angles of binding have been determined by electron microscopy [35], their neutralization profile helps identify the epitopes targeted by the polyclonal sera. The MAbs generally resembled the Group-1 sera in their mutant recognition patterns. But MAb 11B differed in that it was moderately sensitive to the N465-KI mutation, although not to the C3 changes that also define the C3/465 epitope (Fig 3A). Since the glycan knocked-in at residue-465 is quite distant from the 241/289-glycan-hole core epitope for these MAbs, the moderate effect of the N465-KI mutation is plausibly indirect (see Discussion).

### Mapping autologous NAb responses in BG505 SOSIP trimer-immunized macaques

**Fig 6:**
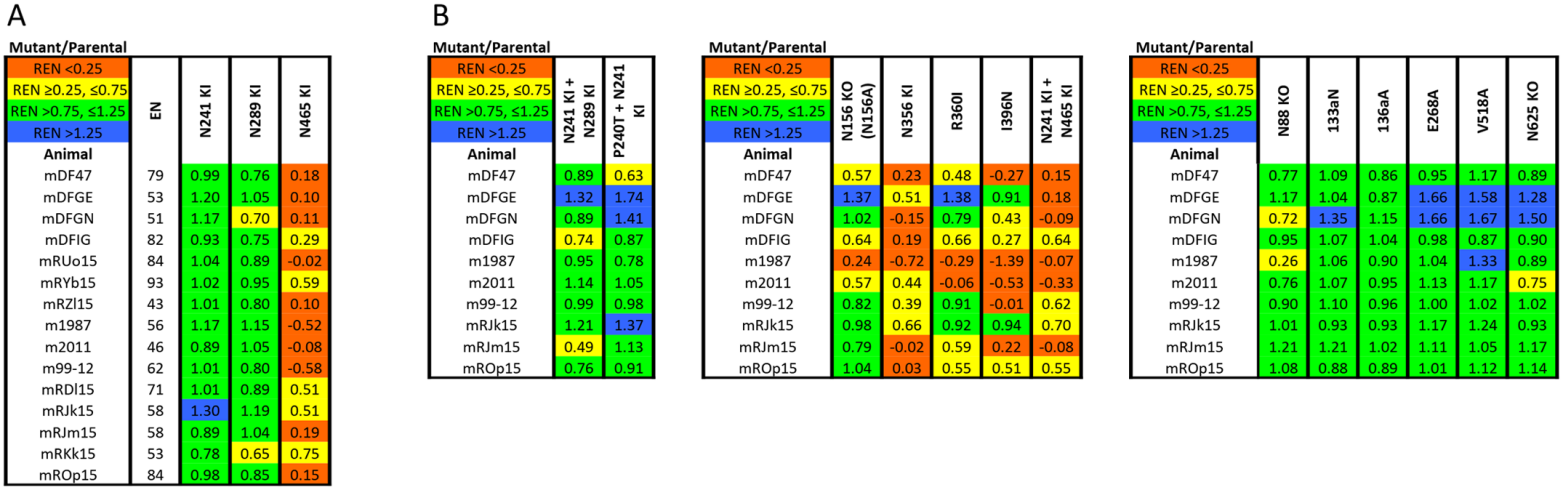
Epitope mapping of autologous NAb responses in BG505 trimer-immunized macaques. Macaque samples (analyzed as purified IgG) are arranged in the left-most column according to the studies they were obtained from. The effect on neutralization is relativized as the extent of inhibition of mutant over that of parental IgG at a concentration corresponding to a 1/50 dilution of serum (REN = relative extent of neutralization, color-coded as in the key at top-left). **A**. Fifteen macaque IgG samples were tested against mutants that most affected neutralization by rabbit sera. The extents of neutralization of the parental virus (EN, %) are given in the first data column. **B**. Ten macaque IgG preparations were tested against the N241-KI/N289-KI and the N241-KI/P240T double mutants as well as selected mutants involving C3, V1 and other residues.

We purified IgG from 15 sera derived from four different exploratory studies of the immunogenicity of BG505 SOSIP trimers in macaques (see Methods). The IgGs were first tested against three key BG505.T332N mutants identified by the rabbit serum analyses outlined above. The N241-KI mutation did not reduce neutralization by any of the 15 IgG preparations, while the N289-KI mutation conferred moderate resistance in only two cases. In contrast, the N465-glycan-KI change reduced the extent of neutralization for all 15 IgG preparations, albeit only moderately in five cases (Fig 6A).

Sufficient IgG was available from 10 monkeys for a more detailed analysis (Fig 6B). The N241-KI/N289-KI double mutant behaved similarly to the two single mutants in that it was moderately resistant to two of the 10 samples, which further indicates that the 241/289-glycan hole is not strongly immunogenic in macaques (Fig 6B). In contrast, the C3 mutations N356-KI and R360I and the V4 change I396N reduced neutralization by all or most of the macaque IgGs; these mutants generally tracked the N465-KI just as they did with the rabbit sera (Fig 6B, compare with Fig 3). For 4 of the 10 IgG preparations, the N241-KI/N465-KI mutation had somewhat less impact than the single N465-KI change (Fig 6B). Furthermore, the N156-KO mutation, which did not reduce neutralization by any of the rabbit sera, did reduce it by 4 of the macaque IgGs (Fig 6B, compare with S1 Fig). The immuno-dominance of the C3/465 epitope over the 241/289-glycan hole was confirmed by the RAUC method, which, however, registered ~10–20% fewer neutralization-reducing effects of C3/465 mutations (S3 Fig). Finally, the various mutations other than N241-KI and N289-KI that reduced neutralization by rabbit Group-1 sera, and the V1 mutations that affected two Group-2 sera, did not markedly reduce neutralization by the macaque IgG preparations (Fig 6B, compare with Figs 2B and 4).

## Discussion

Here we have epitope-mapped the NAb responses to BG505 SOSIP trimers in rabbits and rhesus macaques. We previously reported that the 241/289-glycan hole accounted for the NAb response in over half of the BG505 SOSIP.664 trimer-immunized rabbits [23]. That rabbit MAbs elicited by this trimer bind at this site confirms that this epitope is immunogenic in the rabbit [35]. We can now expand this epitope cluster to the region around residue D230, because knocking in a glycan at this position has nearly the same effect as the N241-KI and N289-KI mutations. Of note is that all rabbits immunized with the clade B trimer B41 SOSIP.664 raised NAbs that were blocked by the N289-KI mutation [23]. By analogy to the BG505-epitope mapping, whether NAbs induced by the B41 trimer are also affected by mutations other than the N289-KI change should now be investigated.

We previously noted that the 241/289-glycan hole was not the entire story: a substantial proportion of the trimer-immunized rabbits raised NAbs that targeted one or more additional epitopes [23]. We now show that one such NAb epitope involves a stretch of the C3 region (around the β14 strand), and that a glycan added at residue T465 strongly shields this epitope. Residue T465 is located at the junction of V5 and the β24 strand [36]. One caveat about the 465-KI mutation is that it can have a modest effect on the 241/289-glycan hole epitope, as judged by data on MAb 11B (Fig 3A). As the C3/465 and 241/289-glycan hole epitopes do not abut (Fig 5B), an indirect impact of the knocked-in glycan on the accessibility or structure of the latter epitope is probably responsible. Such an effect should be borne in mind in the future use of this particular mutant. The mutations in C3, however, lacked these apparent distant effects, and therefore the C3 mutants more definitively characterize the epitope. The contribution of C3 residues to the C3/465 epitope is consistent with the observation that the C3 region was under immune selection pressure in the human infant from whom the BG505 virus was isolated [13, 16], and with our earlier, more limited epitope mapping based on an Env protein with C3-sequence changes (the 7C3-mutant) as a competitor in NAb assays [25]. The C3 region is also the target of early, narrow specificity NAb responses in people infected with clade C HIV-1 strains [37, 38]. Indeed, despite its designation, the C3 region is not strictly conserved in all clades. The original classification of this Env segment as conserved was based on sequence comparisons within clade B, but C3 is more variable in viruses from other clades and may be widely targeted by NAbs during HIV-1 infection [39–42].

The V1 region near residues N133 and N136 was involved in neutralization by two of the 15 rabbit sera tested. This site is probably included in an additional, infrequently immunogenic NAb epitope on the BG505 SOSIP trimer. Its spatial relationship to the other two autologous NAb epitopes is shown in Fig 5B.

As noted earlier, we selected the rabbit sera in Groups-1, -2 and -3 solely to define new epitopes. Therefore, the group sizes do not reflect the proportions of sera with the same properties across a non-selected group. We previously described sera from 30 BG505 SOSIP.664-trimer-immunized rabbits [23]. Applying the current titer-based analysis to these sera suggests that the 241/289-glycan hole is the dominant target in ~50% of the animals and the C3/465 epitope in ~25%; the other 25% of the sera with intermediate profiles may contain NAbs against both epitopes. We found no evidence that NAbs in any of the rabbits are directed solely to V1 epitopes; no matter how they act, the V1 sequence changes affect the overall neutralization capacity in only ~10% of the animals.

We detected marked correlations between the relative titers against the N241-KI or N289-KI mutants and the absolute titers against the parental BG505.T332N pseudovirus. The 241/289-glycan hole-directed NAbs have a severely limited breadth of neutralization against heterologous Tier-2 and -3 viruses because glycans are frequent at these positions [23]. The higher neutralization titers associated with responses to the C3/465 epitope are therefore noteworthy, as conserved elements of this site may represent a more negotiable route to neutralization breadth.

We observed many examples, particularly glycan-KO mutatants, of increased neutralization sensitivity. It is possible that the removal of the glycans, or other sequence changes, permits NAb access to nearby but otherwise cryptic immunogenic epitopes. Or these neutralization-sensitizing mutations may allosterically render the virus more sensitive to NAbs against the 241/289-glycan hole or C3/465 epitopes. Another explanation, however, is that they act by increasing the accessibility of V3 or other epitopes for otherwise non-neutralizing antibodies. But a change in the Tier status of the BG505.T332N virus from Tier-2 to Tier-1 would presumably enhance neutralization by the vast majority of these rabbit sera. Additional studies would be required for a more definitive account.

Our analyses of BG505 SOSIP trimer-immunized macaques reveal that the 241/289-glycan hole is not the major NAb epitope in this species, as it was targeted only in a minority of animals. In an analysis of a different set of sera from various BG505 SOSIP trimer-immunized macaques, N241-KI and N241-KI/N289-KI mutants were moderately resistant in, respectively, one and six out of nine cases (and the N332T mutant in one of nine cases); no other NAb epitope was identified [24]. Differences in immunizations and analyses probably account for the modest differences in the frequency and extent to which the 241/289-glycan hole was apparently targeted in the two studies (see also SI section). Here, we show that the dominant epitope in the macaque is the C3/465 site. The macaque differs, therefore, from the rabbit, not in the identity of the BG505 Env epitopes to which it mounts NAb responses but in the relative frequency with which it does so against the different epitopes. We do not think that such a difference precludes the use of rabbits in preclinical studies of BG505 trimer variants, although, all other things being equal (which, from a practical and financial perspective, they are not), the macaque would be our preferred model. Whether the detailed immunogenicity profiles of trimers derived from other HIV-1 genotypes vary between species remains to be determined; but that they might vary should be borne in mind when animal immunization studies are designed and interpreted. Finally, on the reasonable assumption that, immunologically, humans resemble macaques more than they do rabbits, we propose that the dominant autologous NAb response in BG505 SOSIP.664 trimer-immunized humans will be against the C3/465 epitope. This hypothesis may eventually be testable [29].

We sought improved knowledge of the autologous NAb responses to BG505 SOSIP trimers to facilitate the design of immunogens yielding greater neutralization breadth. During HIV-1 infection, broader responses sometimes evolve over a multi-year period after the initial narrowly specific autologous NAbs that appear in the first few weeks to months. A single immunogen, such as a BG505 SOSIP trimer, may be able to mimic the first stimuli of this complex process but is unlikely to suffice for the induction of more broadly active NAbs. The challenge might then be to mold that initial response towards breadth. Structural knowledge of the different epitopes for trimer-induced autologous NAbs may help in overcoming sequence variation nearby. Alternatively, if an autologous response is not amenable to broadening, it may be fruitful to block the epitope and direct the initial response to epitopes with a greater potential for cross-reactivity. The new information we have obtained on BG505 NAb epitopes may help strategies to accomplish these goals.

## Materials and methods

### Sources of rabbit sera

Female New Zealand White rabbits were immunized intramuscularly with the BG505 SOSIP.664 trimer formulated with 75 Units of Iscomatrix, essentially as described previously [21, 23, 25]. Iscomatrix is a saponin-based adjuvant obtained from CSL Ltd. (Parkville, Victoria, Australia) via the International AIDS Vaccine Initiative [23, 25, 43]. The rationale for the selection of 15 sera for epitope mapping is outlined in Results. The protocols used to obtain these sera varied as outlined below. Rabbits r5723-r5727 [23] were immunized with 30 μg of trimer at weeks 0, 4, and 20 and sera were obtained at week 22. Sera from rabbits r5738-r5742 were obtained at week 62 after immunizations with 30 μg of BG505 SOSIP.664 trimers at weeks 0, 4, 48 and 60 (with intercalated B41 SOSIP.664 trimer at weeks 20, 24 and 36) [23]. The serum from rabbit r5747 was obtained at week 22, after immunizations with 15 μg of each of the BG505 SOSIP.664 and B41 SOSIP.664 trimers at weeks 0, 4, and 20, and from rabbit r5744 at week 26 after such immunizations at weeks 0, 4, 20, and 22 [23]. The latter schedule with BG505 SOSIP.664 and B41 SOSIP.664 trimer doses of 45 μg was used before obtaining week 22 sera from rabbits r5749 and r5751 [23].

Monoclonal antibodies (MAbs) 11A and 11B, isolated from BG505 SOSIP.664 trimer-immunized rabbits, have been described previously [25, 35].

### Sources of macaque sera

The immunization protocols are summarized briefly as follows.

Macaques DF47, DFGE, DFGN and DFIG received 300 μg of BG505 SOSIP.664 trimer in Iscomatrix (75 units) adjuvant intramuscularly at weeks 0, 4 and 20 (DF47 and DFGE had been primed with an adenovirus vector Ad 26 expressing BG505 SOSIP.664 Env at 30 and then 18 weeks prior to week-0). The test sera were obtained at week 22; i.e., 2 weeks after the third trimer immunization. The study was carried out at Alphagenesis Inc., Yemassee, SC.

Macaques RUo15, RYb15 and RZI15 received 100 μg of BG505 SOSIP.664 trimers subcutaneously in the right leg close to the popliteal lymph nodes, in Iscomatrix (75 units) adjuvant at weeks 0, 6, 12 and 24. The test sera from RUo15, RYb15 and RZI15 were obtained at weeks 36, 32 and 26, respectively; i.e., 12, 8 and 2 weeks after the 4th immunization. The study was carried out at the Yerkes National Primate Research Center (NPRC) at Emory University, Atlanta, GA.

Macaques rh1987 and rh2011 received 100 μg of BG505 SOSIP.664 trimers in Iscomatrix adjuvant intramuscularly at weeks 0, 4, 12 and 24. The test sera were obtained at weeks 26 and 28, respectively, i.e., 2 or 4 weeks after the fourth immunization. The study was performed at the Wisconsin NPRC, Madison, WI [25].

Macaques 99–12, RDL15, RJk15, RJm15, RKk15 and ROp15 received two 100 μg doses of BG505 SOSIP.v5.2 trimers, delivered by subcutaneous immunization in the thigh of each leg, at weeks 0, 6, 12 and 18. The adjuvant for RJk15 was Iscomatrix, for the other macaques it was PLGA (MPL+R848). The test sera were obtained at week 20 for RDL15 and RKk15 and at week 21 for 99–21, RJk15, RJm15 and ROp15; i.e., 2 or 3 weeks after the fourth immunization. The study was carried out at the Yerkes NPRC [22].

### Ethics statement

The rabbit immunization experiment from which the serum samples were derived has been described previously [23]. The study was approved and carried out in accordance with protocols provided to the Institutional Animal Care and Use Committee (IACUC) at Covance Research Products (CRP) Inc. (Denver, PA), study number C0014-15.

The macaque immunizations were also carried out according to the NIH guidelines in compliance with IACUC regulations. Approvals were obtained from the Center for Virology and Vaccine Research, Beth Israel Deaconess Medical Center and Emory Vaccine Center for the experiments carried out at Alphagenesis Inc, the Yerkes NPRC and the Wisconsin NPRC respectively (see above).

The rabbits and macaques were housed, immunized and bled at the various institutions listed above, in compliance with the Animal Welfare Act and other federal statutes and regulations relating to animals, and in adherence to the Guide for the Care and Use of Laboratory Animals, National Research Council, 1996.

### Production and characterization of BG505.T332N Env-pseudotyped virus mutants

Mutant BG505 *env* genes containing point substitutions were made as previously described [15, 25, 44]. The BG505.T332N virus with a full-length cytoplasmic tail was used as the parental for mutant design, as it contains a knocked-in N332 glycan to match its sequence with the corresponding SOSIP trimer immunogens [15, 23, 25]. Some mutants were based on various clones of the MG505 virus, which was isolated from the mother of the BG505 HIV-1-infected infant [13, 16]. The infectivities of the various mutant viruses for Tzm-bl cells were determined by titration (S1 Table). Only viruses yielding a luminescence signal of 1 x 10^5^ (relative light units, RLU) at a dilution of 1/20 or higher were used. These mutants ranged in relative infectivity (RLU/(relative viral dose in parts per volume), compared with the parental virus, from 0.1–6.8.

### IgG purification from macaque sera

In preliminary neutralization assays, we found that macaque sera interfered non-specifically, enhancing the infectivity of the murine leukemia virus and most of the HIV-1 pseudoviruses. We therefore purified IgG from macaque sera or plasma to eliminate the interfering factors. This procedure was not necessary with rabbit sera. Briefly, serum or plasma diluted 25-fold in PBS was passed through an equally mixed Protein A (17-5138-01, GE Healthcare) + Protein G Sepharose column (P3296, Sigma-Aldrich), the bound IgG was eluted with glycine, pH 3 and immediately equilibrated with Tris, pH 8. The eluate was diluted 2.5-fold with PBS and spun in Vivaspin 6 (10 kDa cutoff, GE Healthcare) columns thrice. The recovered IgG was reconstituted to the original volume. IgG recovery was measured by ELISA (RSIGGKT-717, Molecular Innovations).

### Env-pseudotyped virus mutants for mapping NAb epitopes

Neutralization of Env-pseudoviruses by sera or purified IgG was quantified in the TZM-bl-cell assay as described previously [15, 25, 44]. Neutralization was defined as the reduction (%) of the infectivity obtained in the absence of serum or IgG. Rabbit sera were titrated in two-fold steps. The dilution factors reducing infectivity by 50% (half-maximal inhibitory dilution factor, ID_50_) were calculated from nonlinear regression fits of a sigmoid function (with maximum constrained to <100% and minimum unconstrained) to the normalized inhibition data in Prism (Graphpad). The ratios of the titers for each mutant virus over the parental BG505.T332N virus (Relative Inhibitory Dilution at 50% neutralization = RID_50_) are reported in the Results section. The ID_50_ values for the rabbit sera against the parental virus were in the range 300–4000.

The macaque IgG preparations generally had lower neutralizing capacity than the rabbit sera against the BG505.T332N parental virus, which in most cases precluded analyses of titer changes against the mutants. We therefore recorded the ratio of the extent of neutralization of a mutant virus, compared with the parental virus, at a macaque IgG concentration corresponding to a 1/50 dilution of serum or plasma. These ratios, i.e., the relative extents of neutralization, REN, are recorded in Results. In addition, because of limiting sample availability, some of the IgG preparations could only be tested against a subset of mutants.

All neutralization measurements are the medians derived from ≥3 experiments. In order to ascertain the accuracy of the results, we compared three methods for quantifying the effects of mutations on neutralization sensitivity (see SI section). They are based on neutralization titer (RID_50_), neutralization extent (REN) at the lowest serum dilution, and the area under the neutralization curve (RAUC) (S1 Fig). Moderate resistance is less frequently detected as REN or RAUC than as RID_50_ (S1–S3 Figs) although meaningful RID_50_ determinations require ID_50_ values > 300 and were thus not applicable to most of the macaque samples. A theoretical account of how the different resistance measurements might relate to the affinity and stoichiometry of binding is presented in the Supplementary results section (S1 Text, pages 1–2). We note that we have previously described a strong correlation between the potency of neutralization of the BG505.T332N virus (as measured by ID_50_) and of binding to the BG505 SOSIP.664 trimer in ELISA (as measured by EC_50_) [15, 23]. By extrapolation, the relative neutralization and binding titers for the virus and trimer mutants should also correlate, but this remains to be verified experimentally.

### Structural modeling

The three-dimensional model of the BG505 SOSIP.664 trimer was based on the PDB-5V8M structure [45]. The gp120 glycans were modeled as Man5 while gp41 glycans were either derived from PDB-5FUU or modeled as Man5 [46]. UCSF Chimera was used for model visualization [47].

### Statistics

To investigate whether different mutations affected the same or disparate epitopes, we performed Spearman rank correlations of absolute and relative neutralization titers of rabbit sera against selected mutants (two-tailed significance tests). RID_50_ values for subsets of rabbit sera were compared by two-tailed Mann-Whitney U tests (all analyses were performed in Prism, Graphpad).

## Supporting information

S1 Text (DOC)

S1 Fig Glycan-KO and –KI mutations that did not frequently reduce neutralization by the rabbit sera. (XLSX)

S2 Fig Non-glycan mutations that did not frequently reduce neutralization by the rabbit sera or are not surface-located. (XLSX)

S3 Fig Comparison of different methods for calculating the effects of mutations on neutralization sensitivity. (XLSX)

S1 Table List of Env mutants tested in neutralization assays. (XLSX)
