# Supplementary material for: Epitopes for neutralizing antibodies induced by HIV-1 envelope glycoprotein BG505 SOSIP trimers in rabbits and macaques
Source: PLoS Pathog. 2018 Feb 23;14(2):e1006913. doi: 10.1371/journal.ppat.1006913 (PMC5841823; doi:10.1371/journal.ppat.1006913)
Supplement: S1 Text — (DOC) [file ppat.1006913.s001.doc]

**SUPPORTING INFORMATION**

Supplementary results

*List of Env mutants*

The complete list of BG505 Env mutants used to analyze neutralization sensitivity is given in S1 Table. The mutants are divided into the following subgroups depending on the origin of and rationale for their designs: alanine-scanning mutants previously described [1]; mutations derived from the maternal virus MG505 cl.A2 and cl.H3; and several mutations based on structural modeling combined with existing knowledge of the role of the 241/289-glycan hole in an epitope cluster recognized by NAbs in many of the rabbit sera [2].

*Methods for analyzing neutralization of virus mutants*

When seeking a second major epitope cluster seen by rabbit NAbs, we selected a method that allows the effects of a mutation on neutralization by relatively high-titer sera to be detected sensitively: this was the RID_50_ procedure, in which the ID_50_ values against the mutant and parental viruses are compared. In contrast, determining the relative maximum extent of neutralization (REN method) allows the strongest, most unambiguous effects of a mutation to be detected. Of the two methods, we could only use REN with the macaque IgG preparations, since their ID_50_ values were mostly too low to allow the accurate quantification of a substantial titer reduction (i.e., > 4-fold, down to an RID_50_ <0.25; color-coded red). Hence, initially, we used the REN method at the highest IgG concentration in an attempt to map macaque NAb epitopes. Although, as noted, that method is the less sensitive, it is the more specific for the strongest reductions in neutralization sensitivity. Therefore, the clear effects of the C3/465 mutations on neutralization by macaque IgG that were detected by the REN method unambiguously implicate the involvement of this region in a NAb epitope cluster.

To investigate the differences between the methods further and to avoid method bias, we made further extensive comparisons. First, we compared the RID_50_ and REN analyses of the rabbit sera (Figures 1-5 and S1-3 Figures), and found that the RID_50_ method detects intermediate effects more often. Thus, the effects of the Group-2 sera on the 241/289-glycan hole mutants fell in the yellow-colored (intermediate) zone with the RID_50_ method but were green (null effect) with the REN method. And the strong reductions of neutralization by the 241-KI/289-KI mutations in Group 1, as well as a subset of those by the C3/465 mutations in Group 3, were also apparent with the REN method.

Next, we analyzed neutralization of the panel of mutants in Figure 6 by both rabbit sera and macaque IgG using a third method, RAUC, which is based on the area under the neutralization curves for a mutant relative to that for the parental virus (S3 Figure). For the rabbit sera the discriminating power of the N241-KI/N289-KI and the C3/465 mutations was maintained. An exception was serum r5751 in Group-3, which showed only a marginally reduced neutralization of the N465 KI mutant by the RAUC method, whereas, in agreement with the REN results, the double N241-KI/N465-KI mutant was completely resistant. As noted in the main section, however, this serum differed from the others in Group 3 in several respects (Figures 1A, 2A and B and 4). Furthermore, the specific reducing effects of the V1 mutations 133aN and 136aA on the neutralization by two sera in Group 2 were evident by the RAUC method.

The RAUC analysis of the neutralization by the macaque sera also corroborated the REN results. The neutralization-reducing effects of the N241-KI and N289-KI mutations alone and in combination, which were negligible by the REN method, were only marginally increased upon RAUC analysis but so were their enhancing effects sporadically (see below); conversely, the detection of reduced neutralization for the C3/465 mutants was of 10-20% lower frequency than by the REN method. Note, however, that the relative intervals for color-coding in RID_50_ and RAUC are the same but they differ from those in REN. All were chosen to catch significant changes in the respective parameters; still, the color criteria are somewhat arbitrary and the numbers contain the real discriminating information. In conclusion, RAUC analysis largely agrees with but complements the other two methods (S3 Figure).

Overall, we suggest that the epitope specificities of polyclonal NAbs are best dissected by using all three methods, or at least REN and RAUC when the titers are too low for RID_50_ analyses. The use of more than one method can be further justified by considering the virological and immunochemical interpretations of the different relative values. Theoretically, RID_50_ is primarily affected by how much a mutation changes the affinity of NAb binding to the mutant compared with the parental Env trimer. At sufficiently high NAb concentrations, ~100% neutralization can still occur and then the REN will not be reduced even when the RID_50_ is much reduced. In contrast, a reduced REN but an unchanged RID_50_, which is rarer than the converse, could occur when the mutation introduces more heterogeneity among the trimers; this could happen, for example, when over half of the virions remain as sensitive as the parental virus while other virions become completely resistant. Further evidence for increased or reduced antigenic heterogeneity within the mutant trimer and therefore virion population, compared with the parental, can be provided by changes in the neutralization curve slopes [3]. The AUC approach would detect any parallel shifts of sigmoid curves and the suppression of maximum neutralization plateaus equally well. Hence, this method would be a more sensitive way to study the combined reductions of NAb potency and efficacy. The complete loss of neutralization detected by all of the methods would not necessarily be attributable to the complete loss of antibody binding, but could arise because the NAbs fail to achieve the required neutralizing occupancies on any virions [4-7]. We also note sporadic enhancement of the infectivity of a mutant by a serum, thus yielding negative REN values, when the parental virus is strongly neutralized. If this enhancement is due to sub-neutralizing occupancies of NAbs [8-10], it does not complicate the interpretations of reduced neutralization. But if it is due to the binding of non-NAbs conferred by the mutation it would complicate them; for it could then counteract simultaneous neutralization. Finally, the RID_50_ and RAUC have more leeway than does REN for detecting enhanced neutralization sensitivity; only when the maximum neutralization of parental virus is low can REN be tangibly increased (see S1-3 Figures).

*Interpretations of the effects of some selected mutants*

We found that the V2 mutation Y177A conferred near pan-resistance to the rabbit sera, while the nearby D167A, K168A and mutations moderately reduced the neutralization potency of Group-3 sera (S2 Figure). The V172A, Y173A, L179A and V181A mutations had mixed effects. Structural modeling shows that none of the hydrophobic residues above (i.e., V172, Y173, Y177, L179 and V181) is located on the trimer surface. These various mutations may act by altering the packing of the surrounding residues or the processing of one or more glycans and hence, indirectly, the exposure or shape of the NAb epitopes. We do not consider them valuable for epitope-mapping, but understanding how they act may be worth additional investigation.

The N611-KO mutation in gp41 has been reported strongly to increase the neutralization potency of the 12A lineage of MAbs, which were isolated from a BG505 SOSIP.664-immunized rabbit [11]. Here, N611-KO mutations had no significant effect on neutralization by any of the 15 rabbit sera (S1 Figure). But our analytical panel did not include sera that barely neutralized the parental virus, and the N611-KO mutation presumably augments neutralization only when weak NAbs like 12A predominate in a serum. The N611-KO mutation may have indirect effects on the 241/289-glycan hole epitope. A resistance mechanism that involves the loss of a single glycan, i.e., from residue N611, might occur relatively easily under *in vivo* conditions.

The 133aN and 136aA mutations, which specifically abrogated neutralization by two Group-2 sera, had the converse effect on some Group-1 sera in that they enhanced neutralization. Three nearby mutations, N130-KI, N133-KO and N142-KO, likewise increased the RID_50_ values for the r5743 and r5744 sera and also for some Group-1 sera (S1 Figure). These findings further reveal that local antigenic conditions can have subtle effects on neutralization. Thus, for the r5743 and r5744 sera, moving the N133-glycan one step C-terminally and knocking it out entirely had opposite effects. The partial similarity between the Group-1 sera and the r5743 and r5744 sera, together with the unique features of the latter two, suggests that these two have at least a dual specificity.

**SI Figure Legends**

**S1 Figure:** **Glycan-KO and –KI mutations that did not frequently reduce neutralization by the rabbit sera**

The sera are ordered as in Figures 1-5. Only glycan-KO mutants, listed sequentially from N- to C-terminus, are shown. Whether any of the enhancing mutations act by sensitizing the virus to otherwise non-neutralizing antibodies (e.g., to the V3 region) that are present in the sera has not been evaluated. Particularly the KO mutations frequently enhanced the neutralization titers of the rabbit sera. The top panels show the relative inhibitory dilution (RID_50_) values and the bottom panels the relative extent of neutralization (REN).

**S2 Figure: Non-glycan mutations that did not frequently reduce neutralization by the rabbit sera or are not surface-located**

The sera are ordered as in Figures 1-5 and SI Figure 1. The mutants are listed from N- to C-terminal. The top panels show the relative inhibitory dilution (RID_50_) and the bottom panels the relative extent of neutralization (REN).

**S3 Figure: Comparison of different methods for calculating the effects of mutations on neutralization sensitivity**

**A.** The relative extent of neutralization (REN) is given for the rabbit sera to complement the relative inhibitory dilution (RID_50_) values in main Figures 1-5 (the mutants appear in the same order and with the same subdivisions as in those figures). **B.** Relative area under the curve (RAUC) for rabbit (with prefix r) and macaque (with prefix m) sera are given with the same order of mutants as in the main Figure 6.

**S1 Table. List of Env mutants tested in neutralization assays**

**REFERENCES**

1. Sanders RW, van Gils MJ, Derking R, Sok D, Ketas TJ, Burger JA, et al. HIV-1 VACCINES. HIV-1 neutralizing antibodies induced by native-like envelope trimers. Science. 2015;349(6244):aac4223. doi: 10.1126/science.aac4223. PubMed PMID: 26089353; PubMed Central PMCID: PMCPMC4498988.

2. Klasse PJ, LaBranche CC, Ketas TJ, Ozorowski G, Cupo A, Pugach P, et al. Sequential and Simultaneous Immunization of Rabbits with HIV-1 Envelope Glycoprotein SOSIP.664 Trimers from Clades A, B and C. PLoS Pathog. 2016;12(9):e1005864. doi: 10.1371/journal.ppat.1005864. PubMed PMID: 27627672; PubMed Central PMCID: PMCPMC5023125.

3. Ketas TJ, Holuigue S, Matthews K, Moore JP, Klasse PJ. Env-glycoprotein heterogeneity as a source of apparent synergy and enhanced cooperativity in inhibition of HIV-1 infection by neutralizing antibodies and entry inhibitors. Virology. 2012;422(1):22-36. doi: 10.1016/j.virol.2011.09.019. PubMed PMID: 22018634; PubMed Central PMCID: PMCPMC3229656.

4. Klasse PJ. Modeling how many envelope glycoprotein trimers per virion participate in human immunodeficiency virus infectivity and its neutralization by antibody. Virology. 2007;369(2):245-62. doi: 10.1016/j.virol.2007.06.044. PubMed PMID: 17825343; PubMed Central PMCID: PMCPMC2317823.

5. Klasse PJ. The molecular basis of HIV entry. Cell Microbiol. 2012;14(8):1183-92. doi: 10.1111/j.1462-5822.2012.01812.x. PubMed PMID: 22583677; PubMed Central PMCID: PMCPMC3417324.

6. Magnus C, Regoes RR. Estimating the stoichiometry of HIV neutralization. PLoS Comput Biol. 2010;6(3):e1000713. doi: 10.1371/journal.pcbi.1000713. PubMed PMID: 20333245; PubMed Central PMCID: PMCPMC2841622.

7. Regoes RR, Magnus C. The role of chance in primate lentiviral infectivity: from protomer to host organism. Prog Mol Biol Transl Sci. 2015;129:327-51. doi: 10.1016/bs.pmbts.2014.10.013. PubMed PMID: 25595809.

8. Klasse PJ. Neutralization of Virus Infectivity by Antibodies: Old Problems in New Perspectives. Adv Biol. 2014;2014(Article ID 157895,):1-24. doi: 10.1155/2014/157895. PubMed PMID: 27099867; PubMed Central PMCID: PMCPMC4835181.

9. Klasse PJ, Burton DR. Antibodies to West Nile virus: a double-edged sword. Cell Host Microbe. 2007;1(2):87-9. doi: 10.1016/j.chom.2007.04.001. PubMed PMID: 18005685.

10. Pierson TC, Diamond MS. A game of numbers: the stoichiometry of antibody-mediated neutralization of flavivirus infection. Prog Mol Biol Transl Sci. 2015;129:141-66. doi: 10.1016/bs.pmbts.2014.10.005. PubMed PMID: 25595803; PubMed Central PMCID: PMCPMC4910618.

11. McCoy LE, van Gils MJ, Ozorowski G, Messmer T, Briney B, Voss JE, et al. Holes in the Glycan Shield of the Native HIV Envelope Are a Target of Trimer-Elicited Neutralizing Antibodies. Cell Rep. 2016;16(9):2327-38. doi: 10.1016/j.celrep.2016.07.074. PubMed PMID: 27545891; PubMed Central PMCID: PMCPMC5007210.
